# Influence of electrode placement on the recognition of different gesture categories using high-density sEMG

**DOI:** 10.3389/fnins.2025.1750792

**Published:** 2025-12-29

**Authors:** Fang Qiu, Xiaodong Liu, Xinming Ye

**Affiliations:** 1 School of Physical Education and Health, Shanghai University of International Business and Economics, Shanghai, China; 2 Institute of Physical Education, Guangxi University of Science and Technology, Liuzhou, China; 3 School of Sports Science and Engineering, East China University of Science and Technology, Shanghai, China

**Keywords:** gesture category, hand gesture recognition, HD-sEMG, human-machine interaction, sampling position, SVM

## Abstract

High-density surface electromyography (HD-sEMG)-based gesture recognition serves as a critical interface for human-computer interaction (HCI). However, recognition accuracy exhibits a significant dependency on gesture complexity and electrode positioning. To address this, we systematically investigated the relationship between gesture types and sEMG electrode placement locations through intra-subject, inter-day, and inter-subject validation protocols. Two distinct gesture categories were analyzed, i.e., single-degree-of-freedom (single-DoF) gestures and daily-used multi-finger synergistic gestures. Using an open-access gesture dataset, HD-sEMG signals were acquired from three forearm regions: the distal wrist, mid-forearm, and proximal elbow, separately. Classification results using support vector machine (SVM) revealed that single-DoF gestures achieved peak accuracy with distal wrist signals (98.63% for intra-subject, 79.73% for inter-day, and 75.47% for inter-subject validation protocols), whereas daily-used gestures performed optimally with signals from the mid-forearm and proximal elbow regions. These findings demonstrate the specific relationship between electrode placement and gesture type, providing valuable insights for EMG-HCI design and sensor placement strategies based on the nature of the target gesture.

## 1 Introduction

As the interface bridging human intentionality and digital systems, Human-Computer Interaction (HCI) has evolved from command-line interfaces to multimodal paradigms focused on intent-driven natural communication (Xiong et al., 2024; Kaifosh et al., 2025). Electromyography (EMG), a physiological electrical signal reflecting muscle contraction activity, has emerged as a promising modality for gesture command recognition due to its direct physiological connection to human motion intent (Dai and Hu, 2019). Unlike traditional interfaces (e.g., buttons, touchscreens, and voice assistants), EMG-based interaction technologies offer significant advantages, such as early recognition of intent, comprehensive physiological feedback, and strong resilience to environmental noise (Zheng et al., 2022). These strengths have facilitated the integration of EMG into commercialized HCI systems (Nozaki et al., 2023), such as the i-Limb prosthesis developed by Touch Bionics, which classifies 26 hand gestures using surface EMG (sEMG) and dynamically adjusts grip force for amputees daily object manipulation (Connolly, 2008). In rehabilitation, the Fleischer Bionic Suits exoskeleton systems use EMG-driven actuators to enhance user strength following spinal cord injury (Xiong et al., 2024). In daily applications, the wearable Myo Armband (Muhammad et al., 2020) and Meta Wristband (Harini and Manoj, 2024) utilize multi-channel EMG for gesture-based control of unmanned aerial vehicles and AR/VR interactions.

Recently, high-density surface electromyography (HD-sEMG) has overcome the limitations of traditional point-sensing techniques (He et al., 2019), with electrode arrays featuring multiple sampling points that map spatiotemporal muscle activation patterns. This advancement enables unprecedented accuracy in complex gesture recognition (Geng et al., 2016; Karrenbach et al., 2022; Guo et al., 2025). Crucially, the core of HCI relies on pattern recognition, which allows digital systems to interpret complex biological signals and translate them into actionable commands (Lu et al., 2017). Pioneering machine learning methods for HD-sEMG-based gesture recognition have demonstrated their superiority in terms of computational efficiency, data minimalism, high interpretability, and robustness (Prabhakar and Won, 2023; Wahid et al., 2018; Challa et al., 2023). Common machine learning approaches include support vector machines (SVM) (Grattarola et al., 2025; Li et al., 2020), linear discriminant analysis (LDA) (Ding et al., 2017), random forests (RF) (Jiang et al., 2024), maximum likelihood estimation (MLE) (Nsugbe et al., 2020), and K-nearest neighbors (KNN) (Agarwal et al., 2024). In Prabhakar and Won (2023), a least-squares SVM with hybrid kernels was explored to improve the nonlinear separability of different gestures. By combining the grasshopper feature selection algorithm with KNN, a 98.5% classification accuracy for six gestures was achieved, with a 75% reduction in feature dimension (Agarwal et al., 2024). To assess the real-time gesture classification capabilities of machine learning, Guo et al. (2015) conducted a systematic comparison between neural networks (NN) and SVM, demonstrating the advantages of conventional machine learning methods in online applications. Later, Sara et al. (2021) further compared the online and offline classification performance of various machine learning techniques, revealing minimal differences between the two settings. Therefore, employing classic machine learning methods in gesture classification remains highly valuable.

Despite significant advances in sEMG-based gesture recognition, it is important to highlight that the reports mentioned above exhibit considerable variability (ranging from 72 to 99% across studies), which can be partly attributed to inconsistent experimental protocols. Two frequently overlooked factors—electrode positioning along the forearm and gesture type selection—inevitably introduce systematic biases that undermine real-world generalizability (Wang et al., 2024; Meng et al., 2022). Specifically, the spatial distribution of forearm muscles creates region-dependent sEMG sensitivity for different gesture types: (1) Electrodes placed near the wrist primarily capture signals from extrinsic hand muscles, yielding high accuracy for wrist-centric gestures (flexion/extension) but poor discrimination of finger-level gestures due to cross-talk noise; (2) Mid-forearm electrodes exhibit a more balanced sensitivity for both wrist and finger gestures (Qing et al., 2021); and (3) Electrodes placed near the elbow can only record limited muscle activity, thereby reducing the ability to discriminate finger-level gestures (Wang et al., 2024). To mitigate the accuracy degradation caused by electrode-gesture mismatches, two critical factors must be considered. First, for scenarios with fixed electrode positioning, classification should prioritize gestures involving muscles that are clearly activated in the corresponding sections. Second, when specific gestures are required, electrode placement must align with the target muscle topography.

Following these two guidelines, this article methodically explores the relationship between electrode position and hand gesture recognition. The main contribution was summarized as follows:

1) We systematically investigated three distinct electrode placements (distal wrist, mid-forearm, and proximal elbow) to elucidate the differential sensitivity of hand gestures.
2) Both the single-DoF gestures for foundational kinematic analysis and daily-used gestures representing real-world interaction patterns were evaluated in this study to quantify the suitable sampling section on the forearm.
3) Not only was the intra-subject gesture recognition experiment conducted, both the inter-day and inter-subject experiments were also conducted to obtain classification models with better generalization.

The rest of this paper is organized as follows: Section 2 introduces the methods and the dataset to be investigated. Section 3 lists the results of hand gesture recognition. Discussion is presented in Section 4. Section 5 concludes this paper.

## 2 Methods

### 2.1 Subject information

In this study, 20 intact subjects participated in the HD-sEMG acquisition experiment. There were 12 males and eight females among them, and the corresponding mean age was 26.6 ± 4.4 years. All subjects were clearly informed of the purpose of the study and the experimental procedures. And informed consent was signed in advance. The ethics committee of Fudan University reviewed and approved this experiment (approval number: BE2035). Detailed information on this acquisition experiment was stated in Jiang et al. (2021a).

### 2.2 Data acquisition

**Figure 1:**
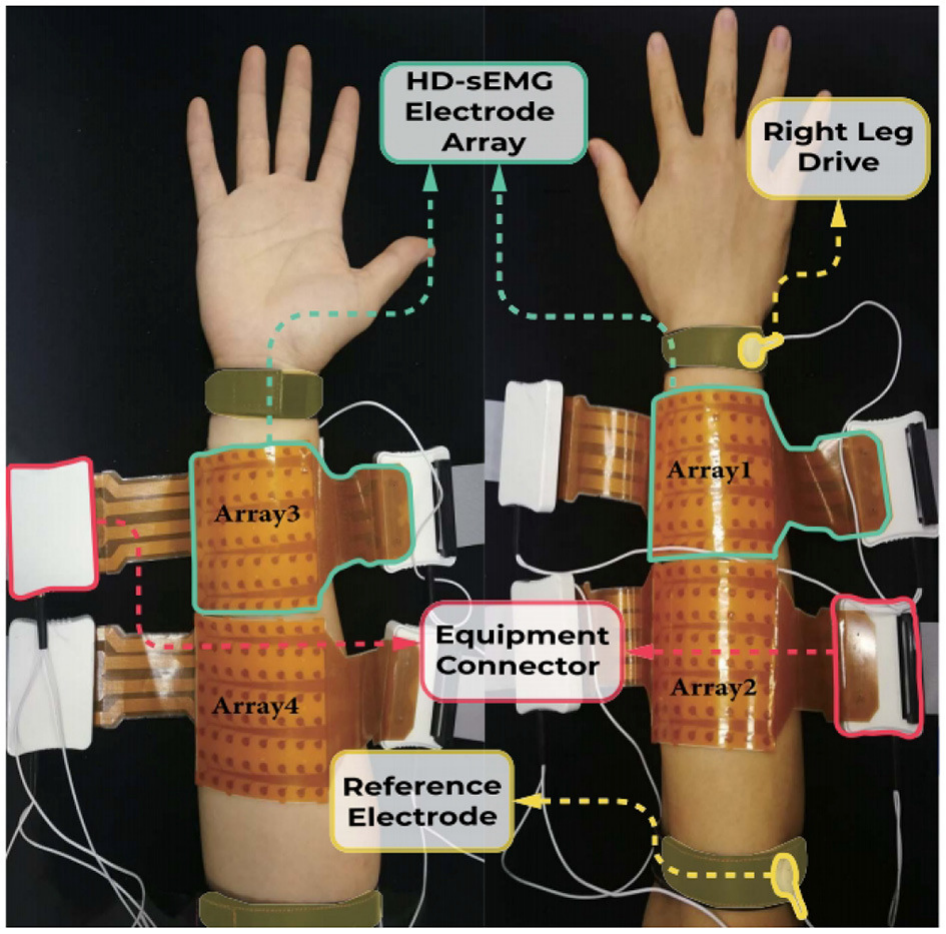
Electrode placement and experiment setup (Jiang et al., 2021a).

After cleaning the subject's right forearm using abrasive gel and alcohol pads, four 8 × 8 electrode arrays (a total of 256 channels) were attached to the forearm, as shown in Figure 1. Each array contained 64 gel-shaped elliptical electrodes for sampling (2.5 mm major axis and 1.4 mm minor axis). The center-to-center distance between two adjacent electrodes in both horizontal and vertical directions was 10 mm. On each side of the forearm, there existed two adjacent arrays, thus forming a 16 × 8 electrode along the long axis of the forearm. To ensure consistent electrode positioning over days, anatomical landmarks were used to demarcate both the flexor and extensor muscle regions (Meng et al., 2022). Corresponding right leg drive and reference electrodes were set on the head of ulna and elbow, respectively. Using the Quattrocento system (OT Bioelettronica, Torino, Italy), the HD-sEMG signals of hand gestures were measured at 2,048 Hz with a band-pass filter ranging from 10 to 500 Hz, a gain of 150, and a resolution of 16 bits.

**Figure 2:**
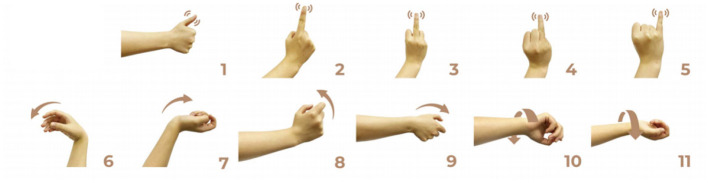
Single-DoF gesture group. The gestures were given as follows: (1) thumb extension, (2) index finger extension, (3) middle finger extension, (4) ring finger extension, (5) little finger extension, (6) wrist flexion, (7) wrist extension, (8) wrist radial, (9) wrist ulnar, (10) wrist pronation, (11) wrist supination.

**Figure 3:**
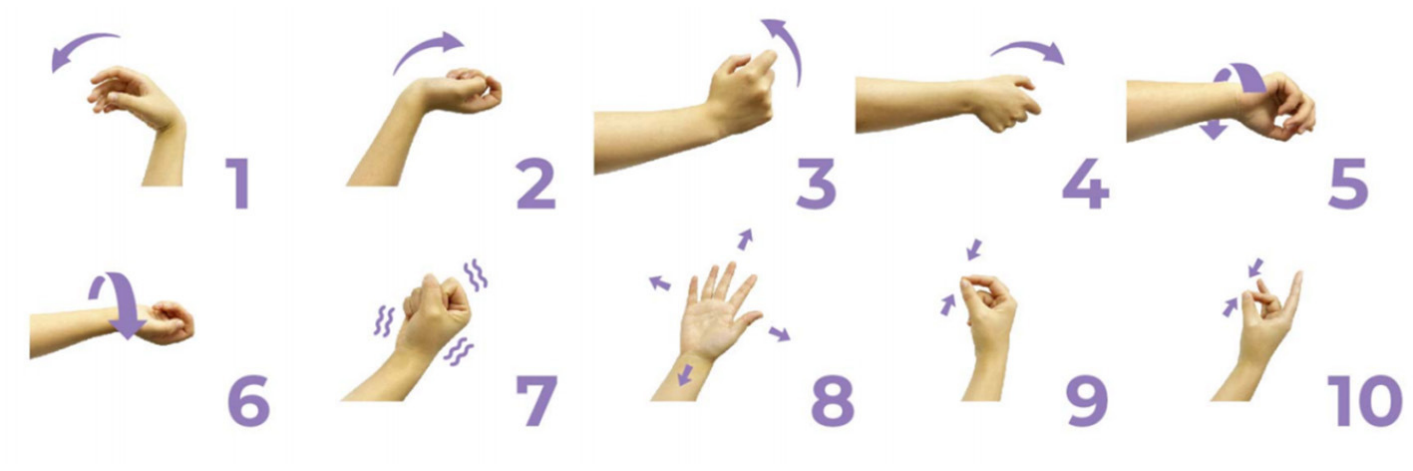
Daily-used gesture group. The gestures were given as follows: (1) wrist flexion, (2) wrist extension, (3) wrist toward radial, (4) wrist toward ulna, (5) wrist pronation, (6) wrist supination, (7) hand close, (8) hand open, (9) thumb + forefinger finger pinch, (10) thumb + middle finger pinch.

During the sampling procedure on a single day, subjects were asked to perform 34 different gestures in a comfortable state, more detail was shown in Jiang et al. (2021a). For each gesture, subjects repeated two trials, with three 1 s dynamic movements included in each trial. To avoid muscle fatigue, a 2 s rest period was provided between movements, and a 5 s rest period was given between trials. On the following day, subjects repeated the same experimental paradigm. In this study, 11 types of gestures involving single-degree-of-freedom (single-DoF) movements were selected for the single-DoF gesture group (shown in Figure 2). These gestures included thumb extension, index finger extension, middle finger extension, ring finger extension, little finger extension, wrist flexion, wrist extension, wrist radial deviation, wrist ulnar deviation, wrist pronation, and wrist supination. Additionally, 10 commonly used gestures were chosen for the daily-use gesture group, including wrist flexion, wrist extension, wrist radial deviation, wrist ulnar deviation, wrist pronation, wrist supination, hand closure, hand opening, thumb and index finger pinch, and thumb and middle finger pinch, as illustrated in Figure 3. Consequently, there were 132 movements (11 gestures × 3 movements × 2 trials × 2 days) in the single-DoF gesture group and 120 movements (10 gestures × 3 movements × 2 trials × 2 days) in the daily-use gesture group for each subject.

### 2.3 Signal processing

The recorded HD-sEMG signals were filtered with a 10–500 Hz eight-order Butterworth band-pass filter. After that, a notch filter combination was employed to attenuate power line interference at 50 Hz and its associated harmonic components up to 400 Hz. Following that, the processed signals were segmented into task-specific epochs for analysis.

#### 2.3.1 Electrode position

**Figure 4:**
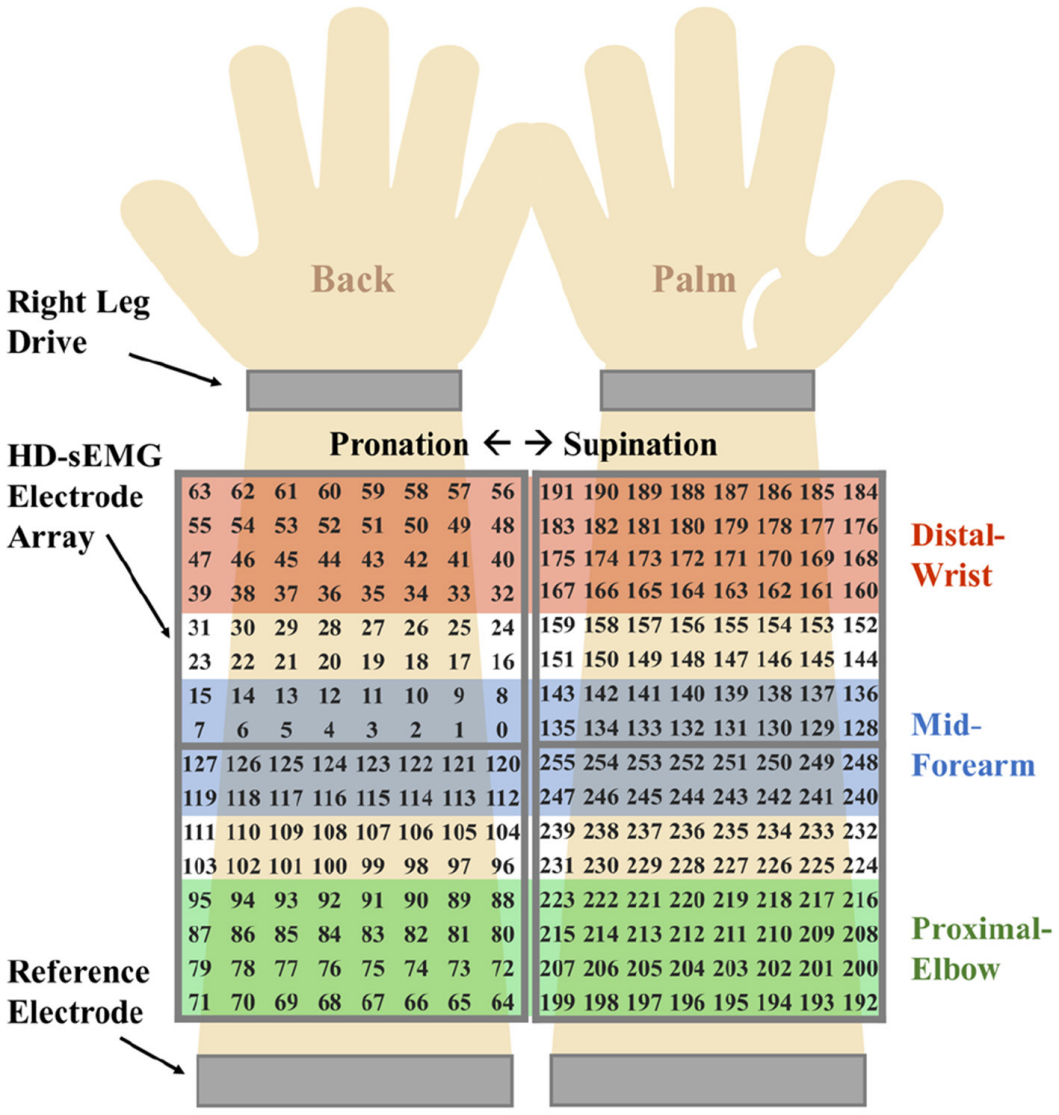
Three electrode placement positions. Red: distal wrist. Blue: mid-forearm. Green: proximal elbow.

To investigate the regional neuromuscular specificity of distinct hand gestures, HD-sEMG signals were recorded from three strategically defined forearm regions (distal wrist, mid-forearm, and proximal elbow) along the proximal-distal axis, as shown in Figure 4. Each region covered a 4 × 16 electrode array.

#### 2.3.2 Window segmentation

For each 1 s gesture movement, the initial 0.25 s reaction phase was excluded. Subsequently, the remaining 0.75 s signal was further partitioned using a 500 ms sliding window with a 125 ms step size (75% overlap), generating three temporally contiguous segments to capture the dynamic evolution of muscle activation while ensuring quasi-stationarity within each window. Subsequently, all the segments were pooled together for feature extraction.

#### 2.3.3 Feature extraction and normalization

For each channel of the HD-sEMG signal, eight distinct features were extracted. The first six features were derived from the temporal waveform of the HD-sEMG signal: Root mean square (RMS) (Jiang et al., 2021b), slope sign change (SSC) (Dai et al., 2017), myopulse percentage rate (MYOP) (Phinyomark et al., 2012), skewness (Skew) (Phinyomark et al., 2013), Willison amplitude (WAMP) (Zardoshti-Kermani et al., 1995), and kurtosis (Kurt) (Phinyomark et al., 2013). The remaining two features characterized the signal in the frequency domain: mean frequency (MNF) (Samaee and Kobravi, 2020) and median frequency (MDF) (Jiang et al., 2020). Based on previous studies (Jiang et al., 2022), the threshold values for MYOP, WAMP, and SSC were set to 0.05 V, 0.05 V, and 0.00004, respectively. This process resulted in a 512 length feature vector for each individual gesture movement recorded at a specific electrode position. Subsequently, all feature vectors underwent *Z*-score normalization for standardization.

#### 2.3.4 Outlier detection and processing

Channel outliers in HD-sEMG, caused by abrupt baseline drift or poor electrode-skin contact, significantly degrade gesture classification performance. To mitigate such signal corruption, a channel outlier reconstruction scheme was implemented. Specifically, for each gesture movement, the 64 feature values extracted from the selected HD-sEMG array were reshaped into a 16 × 4 spatial map, aligning with the physical electrode configuration. Channel outliers were then identified using a *mean* ± *2* × *std* criterion, where *mean* and *std* represented the mean and standard deviation of each individual feature map, respectively. Feature values exceeding the range [*mean – 2* × *std, mean + 2* × *std*] were flagged as outliers and replaced with the mean value of their nearest valid neighboring channels before normalization.

#### 2.3.5 Feature optimization

Principal component analysis (PCA) (Zhang et al., 2014) was utilized to reduce the high dimensionality of feature vectors and accelerate the model training process. It is worth noting that the reduced dimensionality *D* must satisfy the dual constraint:


$$\begin{array}{l} D\leq N_{f}\;\text{and}\;D\leq N_{s}-1 \end{array}$$ (1)

where *N*_*f*_ denotes the length of each feature vector (512) and *N*_*s*_ represents the number of training samples. In this work, PCA was applied after feature normalization. For each subject and protocol, the retained number of principal components *D* was chosen such that (1) *D*<*N*_*s*_−1; and (2) the components jointly explained at least 95% of the variance in the training data. The same projection matrix was used to transform both training and test segments.

### 2.4 Validation protocols

To comprehensively investigate the impact of HD-sEMG measurement locations on gesture classification efficacy, both the single-DoF and daily-use gesture groups were evaluated using HD-sEMG signals sampled from three different forearm sections: distal wrist, mid-forearm, and proximal elbow. Classification efficacy was assessed under three validation protocols:

1) *Intra-subject validation*: for each subject, gesture recordings from a single day were segmented into multiple overlapping windows. All windows originating from the same gesture movement were kept together to avoid temporal leakage. A leave-one-movement-out scheme was used: in each fold, one movement (containing its three segmented windows) served as the test set, and the remaining movements were used for training. This procedure was repeated until every movement had been used once as the test unit. The final intra-subject accuracy for each subject was obtained by averaging across all folds and then further averaged across the two recording days.
2) *Inter-day validation*: to assess cross-day generalization, the complete set of segmented windows from day 1 was used for model training, while all windows from day 2 were used exclusively for testing, ensuring strict temporal separation between training and testing data. This train–test assignment was performed independently for each subject, yielding 20 inter-day accuracy scores.
3) *Inter-subject validation*: inter-subject generalization was evaluated using a leave-one-subject-out approach. In each fold, day 1 data from one subject were held out as the test set, while day 1 data from the remaining 19 subjects constituted the training set. No subject appeared in both training and testing sets within the same fold. The process was repeated until all 20 subjects had served as the test subject once, and the final accuracy was averaged across folds.

Support Vector Machines (SVM) (Wang et al., 2023; Kim et al., 2021) were adopted as the classification algorithm due to their demonstrated superiority in handling high-dimensional HD-sEMG feature spaces while maintaining computational efficiency. In every training task, SVM used a one-versus-all coding design to build a multiclass model (Shahzad et al., 2019). linear kernel was used for all SVM classifiers. The hyperparameters *C* and γ were optimized using a grid search with 10% validation on the training set only, ensuring that no test data were used during parameter tuning. The search ranges were *C*∈{0.1, 1, 10} and γ∈{10^−2^, 10^−1^, 1}. The optimal pair was selected based on mean validation accuracy.

### 2.5 Statistical analysis

Because classification accuracy is bounded and deviates from normality, parametric repeated-measures ANOVA was not applied. Instead, statistical significance across the three electrode regions was evaluated using the Friedman test for repeated measures. When the omnibus test reached significance, pairwise differences were analyzed using the Wilcoxon signed-rank test with Bonferroni correction. *p* ≤ 0.05 represents significance.

## 3 Results

### 3.1 Classification results for single-DoF gestures

**Figure 5:**
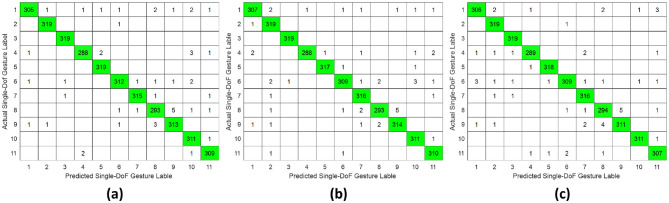
Confusion matrix of single-DoF gesture classification under intra-subject validation protocol accross different forearm regions: **(a)** distal wrist, **(b)** mid-forearm, and **(c)** proximal elbow.

**Figure 6:**
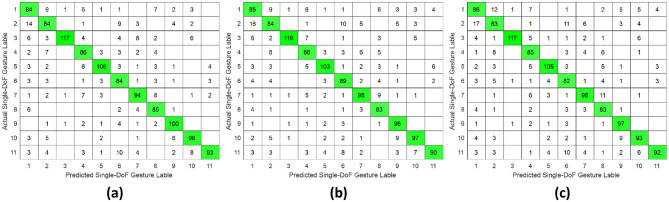
Confusion matrix of single-DoF gesture classification under inter-day validation protocol accross different forearm regions: **(a)** distal wrist, **(b)** mid-forearm, and **(c)** proximal elbow.

**Figure 7:**
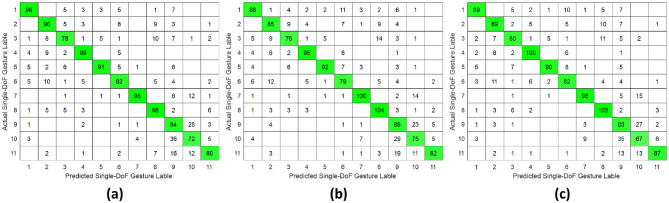
Confusion matrix of single-DoF gesture classification under inter-subject validation protocol across different forearm regions: **(a)** distal wrist, **(b)** mid-forearm, and **(c)** proximal elbow.

Comprehensive results for single-DoF gesture classification under three validation protocols were compiled in Table 1 and Figures 5–7, utilizing HD-sEMG data acquired from distinct forearm regions. The intra-subject protocol yielded the highest accuracy of 98.63%, attributable to a stable electrode-skin interface and consistent neuromuscular activation patterns within the same sampling day. In contrast, the inter-subject validation demonstrated the lowest performance at 75.24% compared with intra-subject and inter-day (*p* ≤ 0.05), primarily due to anatomical variations across subjects, such as wrist pronation and middle finger extension (*p* ≤ 0.05).

**Table 1:** Mean classification accuracy of single-DoF gestures using HD-sEMG data acquired from different regions.

**Location**	**Intra-subject validation**	**Inter-day validation**	**Inter-subject validation**
Distal wrist	98.63%	79.73%	75.47%
Mid-forearm	98.63%	79.42%	75.42%
Proximal elbow	98.51%	78.95%	75.24%

Besides, a consistent trend emerged where classification accuracy degraded as the sampling position moved proximally away from the wrist toward the elbow. Specifically, the poorest results for all three protocols were observed at the proximal elbow region, where reduced muscle belly density and dominant tendon structures diminish sEMG signal quality.

### 3.2 Classification results for daily-used gestures

**Figure 8:**
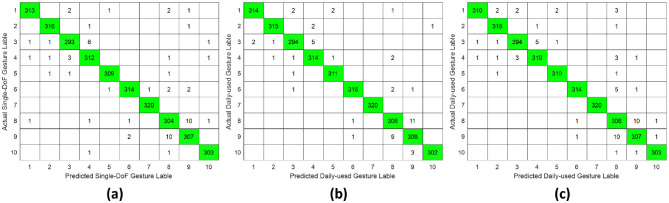
Confusion matrix of daily-used gesture classification under intra-subject validation protocol accross different forearm regions: **(a)** distal wrist, **(b)** mid-forearm, and **(c)** proximal elbow.

**Figure 9:**
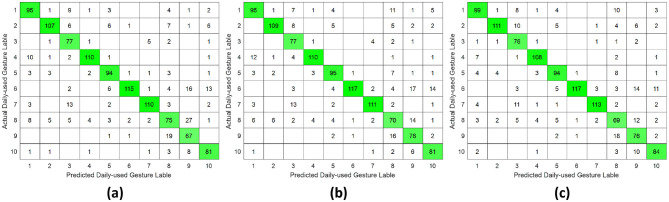
Confusion matrix of daily-used gesture classification under inter-day validation protocol accross different forearm regions: **(a)** distal wrist, **(b)** mid-forearm, and **(c)** proximal elbow.

**Figure 10:**
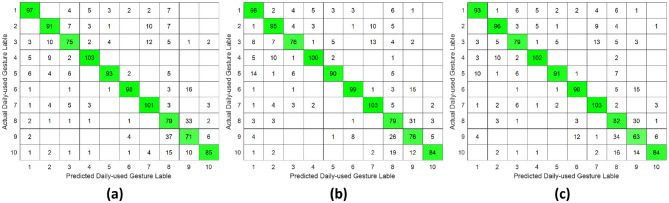
Confusion matrix of daily-used gesture classification under inter-subject validation protocol across different forearm regions: **(a)** distal wrist, **(b)** mid-forearm, and **(c)** proximal elbow.

The classification results for daily-used gestures were summarized in Table 2 and Figures 8–10. Consistent with the findings for single-DoF gesture tasks, the highest mean recognition accuracy (98.37%, *p* ≤ 0.05) was achieved under the intra-subject validation protocol, while the lowest accuracy (75%, *p* ≤ 0.05) occurred in the inter-subject protocol. Inter-day validation accuracy remained comparable to that of single-DoF gesture task, at 80% approximately.

**Table 2:** Mean classification accuracy of daily-used gestures using HD-sEMG data acquired from different regions.

**Location**	**Intra-subject validation**	**Inter-day validation**	**Inter-subject validation**
Distal wrist	98.05%	79.21%	75.22%
Mid-forearm	98.37%	80.20%	75.61%
Proximal elbow	98.21%	80.58%	75.00%

However, unlike the single-DoF gesture classification result, the highest accuracy across all three validation protocols was no longer attained using distal wrist EMG signals. Instead, optimal performance was observed with signals acquired from either the mid-forearm or the proximal elbow. This shift in optimal signal location might stem from daily gestures engaging a broader range of forearm musculature, particularly in the middle and proximal regions, rather than primarily relying on wrist-associated muscles.

## 4 Discussion

This study systematically investigated the relationship between hand gesture recognition performance and electrode placement across three distinct forearm regions: distal wrist, mid-forearm, and proximal elbow, using a publicly available 256 channel HD-sEMG dataset. Two functionally distinct gesture groups were selected. The single-DoF gesture group primarily involved isolated finger movements, while the daily-used gesture group mainly consisted of compound hand movements. For each gesture group, preprocessed HD-sEMG signals were extracted from 64 channel electrode arrays positioned at the three target forearm regions. The subsequent signal processing pipeline included window segmentation, feature extraction, normalization, outlier smoothing, and PCA dimensionality reduction. The resulting feature vectors were then classified using an SVM under intra-subject, inter-day, and inter-subject validation protocols. The overall hand gesture classification results are presented in Tables 1, 2 and Figures 5–10.

This study revealed a relationship between electrode placement along the forearm and gesture classification efficacy, which was highly dependent on the gesture type. Specifically, single-DoF gestures involving finger movements exhibited optimal accuracy when signals were acquired from the distal wrist region, while electrodes placed near the proximal elbow yielded inferior results. These motions, which rely on fine motor control dominated by distal muscles such as the flexor digitorum superficialis and extensor digitorum communis, are densely concentrated near the wrist, ensuring a high signal-to-noise ratio (SNR) in HD-sEMG when sampled from this location (Dai et al., 2019). Conversely, for daily-used gestures involving multi-finger and wrist composite movements (e.g., hand closing or thumb and index finger pinch), this trend reversed, with the proximal elbow sites achieving peak performance. These gestures involve muscle synergies primarily dominated by the wrist and elbow. During these movements, muscles such as the flexor carpi ulnaris and extensor carpi radialis longus at the elbow were activated with high-amplitude EMG signals, alongside the flexors and extensors at the wrist, which provided supplementary dynamic information (Islam et al., 2024). Therefore, it is reasonable that daily-used gesture recognition performed better with HD-sEMG signals from the elbow region. Although Figures 2, 3 represent two gesture sets—single-DoF gestures and daily-used gestures—some gestures across the two sets naturally exhibit overlap because they share similar underlying motor patterns. Single-DoF gestures isolate movement along a specific anatomical degree of freedom (e.g., pure wrist flexion or pronation), whereas daily-used gestures are performed in a natural and comfortable manner and often involve multiple degrees of freedom acting simultaneously. As a result, daily-used gestures may partially recruit the same muscle groups as their corresponding single-DoF counterparts. This leads to similar HD-sEMG activation patterns between the two sets and explains the observed overlap in Figures 2, 3. Therefore, the overlap does not indicate a flaw in class definition; rather, it reflects the inherent neuromuscular relationships and shared muscle activations between isolated and naturalistic gestures.

For the three validation protocols presented in Tables 1, 2, it is evident that the best classification performance occurred in intra-subject validation (around 98%), followed by inter-day validation (about 79%), with inter-subject validation exhibiting the worst performance (around 75%). The superior performance under intra-subject validation can be attributed to the high signal homogeneity, resulting from consistent electrode-skin impedance, stable muscle fatigue conditions, and steady sampling conditions within a single day for each subject. In the case of inter-day classification, despite the efforts to mark landmarks in our experiment, the unavoidable micro-variations in sensor placement altered the signal amplitude and spectral properties. Additionally, factors such as corrupted channels, hardware equipment changes, and environmental noise may vary over time. These unavoidable factors made gesture identification more challenging in inter-day validation, leading to lower classification accuracy (Guo et al., 2024). Beyond these challenges in inter-day classification, the anatomical heterogeneity (muscle volume, fat thickness, forearm morphology, etc.) and neuromuscular movement specificity across different subjects further complicated gesture classification, resulting in the poorest performance in inter-subject validation (Meng et al., 2022). Although the mean accuracies across the three electrode regions appear numerically close, the gesture-specific results reveal substantial class-dependent variability that the overall averages obscure. Distal-wrist signals consistently provide better separability for fine finger-related single-DoF gestures, whereas mid-forearm and proximal-elbow signals more clearly discriminate daily multi-finger synergies. These differences arise from underlying neural-control mechanisms: single-DoF movements are driven by relatively localized and cortically separable motor commands, while naturalistic daily-use gestures recruit distributed cortical–spinal synergies that coordinate both proximal and distal muscle groups. Consequently, distal electrodes capture isolated finger activations more effectively, whereas proximal electrodes better represent multi-joint neuromuscular synergies. Thus, the true influence of electrode placement is reflected not in global accuracy alone, but in systematic shifts in gesture-level separability that align with the hierarchical organization of motor control.

From a neuroscientific and clinical perspective, the identified relationships between electrode location and gesture category have important implications for patient populations such as amputees, stroke survivors, and individuals with spinal cord injury. Distal muscle groups are typically more affected by upper-motor-neuron lesions, while proximal muscles are often relatively preserved. Our findings suggest that gesture recognition systems may need to prioritize proximal-electrode configurations for patients with distal muscle impairment, while distal placements may remain optimal for individuals retaining fine finger control. These insights can guide practical electrode placement for myoelectric prostheses, orthotic exoskeletons, and rehabilitation interfaces.

In contemporary wearable technology, gesture-controlled products utilizing EMG-based classification have become prevalent in daily life. Two dominant form factors have emerged: wristwatch-style devices and armband-style devices, each offering distinct biomechanical advantages for gesture interaction. For wristwatch-style devices positioned at the distal wrist region, optimal performance is achieved with single-DoF micro-gestures (e.g., thumb-index pinch) and simple wrist motions (e.g., radial/ulnar deviation). This preference arises from their proximity to fine-motor muscles, such as the flexor digitorum superficialis and extensor digitorum communis, which enable high-fidelity signal capture for isolated digit movements (Su et al., 2023). In contrast, armband-style devices positioned at the proximal elbow region leverage high-amplitude synergistic signals from muscles such as the flexor carpi ulnaris and extensor carpi radialis longus. These muscles are involved in compound force generation, making armbands ideal for classifying multi-finger dynamic gestures, such as the “OK” sign or hand open/close (Meyers et al., 2024). Critically, for individuals with disabilities affecting hand movements, electrode placement must be customized according to their specific effective gestures.

## 5 Conclusion

In this work, the relationship between hand gesture recognition and EMG electrode positions was explored under intra-subject, inter-day and inter-subject validation protocols. As a result, the HD-sEMG signals from the distal wrist region achieved the best identification for single-finger movement gestures and simple wrist motion gestures yet performed the worst for gestures involving multi-finger actions. In contrast, HD-sEMG signals at the proximal elbow region exhibited the opposite pattern. The classification performance of the HD-sEMG signals sampled at mid-forearm was between the two. Such findings disclosed the sensitivities of different gesture types to HD-sEMG signals from various forearm sections, guiding us in selecting proper sampling positions or gestures under different circumstances. Future work will focus on translating the electrode gesture mapping framework into practical neuroprosthetic and rehabilitation applications. In closed-loop myoelectric prostheses, the identified region-specific advantages (distal for fine single-DoF control, proximal for multi-joint synergies) can guide optimized electrode placement and adaptive decoders that dynamically adjust the weighting of distal versus proximal muscle signals based on the intended gesture. In neuromusculoskeletal modeling, incorporating region-dependent EMG quality may improve muscle activation estimation and torque prediction, enabling more accurate subject specific models for amputees, stroke survivors, or individuals with spinal cord injury. In rehabilitation, the framework can support adaptive training protocols by selecting gesture sets that match preserved neuromuscular pathways for example, emphasizing proximal muscle gestures in early recovery and gradually reintroducing distal gestures as voluntary control improves. These integrations may ultimately enhance personalization and long-term stability in EMG-driven neuroprosthetic and rehabilitation systems.

## Data Availability

The datasets presented in this study can be found in online repositories. The names of the repository/repositories and accession number(s) can be found in the article/supplementary material.
